# Exploration or exploitation? A study on equity incentive design, dynamic decision making, and economic consequences

**DOI:** 10.1371/journal.pone.0277965

**Published:** 2023-01-23

**Authors:** Qianqian Zhang, Chunzi Jiang, Xiaomei Zhang

**Affiliations:** 1 School of Accounting, Southwestern University of Finance and Economics, Chengdu, China; 2 School of Finance, Southwestern University of Finance and Economics, Chengdu, China; Prince of Songkla University, THAILAND

## Abstract

We examine whether equity incentive can encourage exploratory innovation from the perspective of dynamic innovation decision-making process. Using the data of equity incentives in China’s listed companies from 2006 to 2017, we construct exploratory intensity of innovation strategy and analyze the impact of equity incentive on corporation exploratory innovation strategy from both the cross-sectional and time-series perspectives. We find a positive relationship between the vesting period and explorative innovation strategy in the cross-sectional dimension. However, the time-series analyses show that the innovation strategy becomes less explorative and more exploitative after the third period during equity incentive. The effect of vesting period is stronger in smaller firms and during the non-financial crisis period. Further analysis reveals that followed by the changes in innovation strategy, the growth rates of innovation output and firm performance also decline.

## 1. Introduction

Patents are created in substantially different qualities, and high-quality patents are crucial for a firm’s core competency development and long-term performance. As innovation projects are unpredictable, long-term, and high profitable, Manso [1] uses Bayesian decision models and points out that companies should tolerate failure in the short run and award for success in the long run to motivate innovation. Prior literature usually takes the equity vesting period as a whole and found a positive effect of equity incentive plans on innovation [2–4], but less is known about the innovation strategy, which makes it ambiguous about the risk-taking and decision-making behavior of firms during the innovation process. In this paper, we examine the role of vesting period, a key ingredient of the equity incentive plan, in encouraging corporate innovation quality. Specifically, we focus on the exploratory intensity of innovation strategy and identify dynamic corporate decision-making while implementing equity incentive plans.

As an essential channel for firms to determine their innovation direction, innovation strategy is generally categorized into exploratory innovation and exploitative innovation [5]. Exploratory innovation focuses on unknown fields, creates new products or opportunities for corporations, and exploitative innovation includes things as refinement and extension of existing technologies [6, 7]. Exploration usually requires longer research time than exploitation. While exploratory innovation has a high risk [8], it can improve firm competitiveness and produce more novel innovations [9, 10]. Uotila et al. [11] find that there is a trade-off between exploration and exploitation in resource allocation. But less is known about the motivation and process of conducting exploratory innovation. In this paper, we focus on one important element in motivating innovation—equity incentive.

Prior studies have found that equity incentive have a positive effect on corporate innovation [4, 12]. And incentive schemes with long vesting periods lead to better innovation performance [13, 14]. Current literature usually uses R&D investment and patent output to measure innovation, but these measures have significant limitations in measuring innovation [15–17]. Manso et al. [17] argue that R&D expenditures cannot capture the different dimensions of a firm’s innovation strategies. Moreover, patent output only reflects the innovation results in one way, and the patent quality may vary dramatically within and across firms [18]. Our paper fills this gap by studying the relationship between equity incentive and innovation strategy and provides a more detailed analysis of innovation process.

In this paper, we examine the effect of vesting period on corporate innovation strategy in both cross-sectional and time-series views. We use a unique sample of listed Chinese firms because China is currently under innovation transformation and promoting high-quality and high-originality innovation development vigorously. Besides, the implementation of equity incentive was regulated by China Securities Regulatory Commission in December 2005 and has been gradually used by enterprises since then. Compared to U.S. and other developed countries, China has a shorter period of carrying out equity incentives and lacks experience. Lastly, patent quantity cannot fully depict the patent quality situation in developing countries. Therefore, there are great limitations in examining the effectiveness of equity incentives only from the perspective of patent quantity. Identifying innovation strategy and innovation quality is of great importance for developing countries to make better equity incentive regulations.

How does the equity incentive influence corporate innovation strategy? We expect both vesting periods and different phases of equity incentive affect exploratory innovation intensity. Starting from the cross-sectional angle, a long vesting period provides a long research time for exploratory innovation, which is consistent with the fundamental characteristics of exploration [7]. In addition, managerial myopia and executive turnover may decrease when the equity duration is longer [19], and corporate tolerance for innovation failures increases as vesting periods prolong [20], thus motivating the managers to carry out exploratory innovation strategies. However, from the time-series perspective, the persistence of exploratory intensity may change during the equity incentive period. The changes in strategic planning, personnel arrangements, and other potential factors during equity incentives may increase the uncertainty of exploration intensity [5]. Besides, when firms get innovation results after 2–3 years of research, they tend to stop and enter a “harvest season” [21, 22].

We test the above conjecture by using the detailed data of equity incentive plans in China’s listed companies from 2006 to 2017. We find that vesting period is significantly and positively related to exploration innovation strategy. However, the motivating effect decreases as the equity incentive plan proceeds, resulting in a decline in innovation exploration and a rise in innovation exploitation. Following Jia [8], we calculate the innovation strategy as the citation repetition rate, which is the citations used in the patent application year divided by the firm’s existing citation base over the past three years. Our main proxies for the vesting period are the duration of the equity incentive plan and a set of corresponding indicator variables based on each phase of the equity incentive plan.

However, there is a reverse causality problem, as innovation strategy can in turn affect the setting of vesting period. For example, when firms focus on explorative innovation, they may design equity incentives with longer duration to ensure sufficient research time. Moreover, the omitted variable problem may exist though we have controlled a number of variables that may influence innovation strategy. To address these endogeneity challenges, we perform a 2SLS regression using an instrumental variable and propensity score matching (PSM) approach. We also conduct additional tests to make sure our results are robust to alternative model specifications and variable definitions.

We also posit that firm size and external environment may affect the relationship between vesting period and innovation strategy. We find that the positive effect of vesting period is stronger in small firms from the cross-sectional view. However, the time-series analysis shows that large firms perform a more persistent exploration intensity during equity incentive plans. We also examine the influence of financial crisis and show that the association between vesting period and exploratory innovation is more prominent during the non-financial crisis period.

Finally, we focus on the ultimate purpose of equity incentive—patent output and corporate value by examining whether they are affected by the changes in innovation strategy. The analyses reveal that when the innovation strategy becomes less explorative, the growth trends of the company’s innovation output and corporate value decline. This finding indicates that the decrease in exploratory innovation intensity reduces the innovative products output, which hinders the development speed of enterprises.

Our paper contributes to the extant literature in three ways. First, this paper enriches the literature on corporate innovation by providing evidence from corporate innovation strategies. Previous studies have explored the effect of equity incentive on corporate innovation from the aspects of innovation input and output [2, 3]. Different from the previous work, we explore the decision-making process in innovation by measuring the exploratory innovation intensity, which captures the characteristics of innovation projects from the perspective of risk and profit. Second, previous studies mainly emphasize the general effect of equity incentives on innovation [4, 12, 23]. For example, Ederer and Manso [4] find that incentives schemes that tolerate early failure and reward long-term success is effective in motivating innovation. While we focus on the dynamic decision-making process of innovation strategy during every stage of the equity incentive plan. From the cross-sectional and time-series dimension, our work analyzes the companies’ choices and preferences for innovation strategy under different vesting periods and different equity incentive phases, aiming to provide a more comprehensive and profound understanding of corporate innovation. Third, our paper sheds light on the design of equity incentive that may be optimal for promoting firm development. Hellmann and Thiele [24] show that while long-term equity incentive may motivate innovation, it also impairs the incentives for standard projects. We demonstrate that overlong equity vesting periods decrease the growth rates of corporate innovation and firm value through innovation strategy, providing empirical evidence for designing reasonable equity incentive plans to achieve sustainable innovation and developing future corporate performance.

The remainder of the paper proceeds as follows. Section 2 reviews the related literature and develops the hypotheses. Section 3 discusses the sample and variables. Our main empirical results and additional results are presented in Section 4 and Section 5, respectively. Section 6 concludes the paper.

## 2. Related literature and hypotheses development

### 2.1. Related literature

Our paper is related to the literature on equity incentive and corporate innovation. Compared to existing projects, innovation projects are risky, unpredictable, and long-term [25]. Thus, the executives prefer short-term investment because of the threat of poor performance and career concerns [26]. Manso [1] embeds a bandit problem into a principle-agent framework and uses Bayesian decision model to discuss the optimal incentive scheme to motivate innovation. He finds the optimal compensation that motivates innovation involves tolerance for early failure and reward for long-term success. Specifically, an incentive scheme that combines stock options with long vesting periods, golden parachutes, and other job security plans can significantly motivate innovation.

Motivated by Manso [1], prior studies have documented that compared to traditional compensation contracts, equity incentives can motivate executives to carry out innovation activities [4, 12, 23]. A longer vesting period can reduce managerial myopia behavior because it lowers the performance pressure for executives, and tightens the alignment of interests between executives and shareholders. Therefore, a longer equity duration may motivate them to choose an innovation strategy with higher risk, higher return, and longer research time [19, 27]. Moreover, long-term equity incentives increase the enterprise’s tolerance for innovation failure [20, 28], thus ensuring the continuity of R&D investment and innovation output.

However, prior studies have not reached a consensus on whether vesting periods can induce innovation. Some researchers believe that long-term compensation contracts, combined with golden parachutes and other interest-related protection measures for top executives, are more effective in stimulating R&D expenditure and innovation output [13, 14]. In contrast, others find that implementing long-term incentives is a double-edged sword [24, 29]. The sustainable development of a company requires a balance between innovative and non-innovative projects, but encouraging executives to carry out innovation will result in reductions in the investment of existing mature projects.

Another stream of literature explores different innovation strategies. It is crucial for firms to choose a proper strategy for development. Previous studies have documented different methods for effective strategy design under different circumstances, including multi-attribute decision-making [30–32], multi-criteria decision-making [33–35], and sequential decision-making [36]. Following their work, studies have tried to apply these methods in corporate innovation decisions [37, 38]. Given that innovation is highly uncertain [1], the decision-making process of innovation strategy is usually dynamic and may vary over time.

Therefore, existing literature categorizes innovation strategies from different attributes. First, innovation strategies can be divided into product innovation and process innovation according to the supply-demand relationship. Empirical studies find that process and product innovation improve firm performance [39, 40]. Second, as the competition among firms intensifies, the increasing discussions about whether to choose proactive and open innovation strategies for companies has not reached a consensus [41–43].

Third, as different innovation projects have different risks, profitability, and R&D cycle time, prior studies have identified two types of innovation strategy: exploratory innovation and exploitative innovation [5, 6]. Exploitation includes things as refinement and extension of existing technologies. Exploration focuses on unknown fields and creates new products or opportunities for corporations. Compared to exploitation, exploration requires a higher tolerance for failure and thus takes a longer research time. Hirshleifer et al. [16] propose that firms with higher originality innovation experience more persistent and less volatile profitability and higher market values. While Yu and Lee [44] find that the intensity of exploratory innovation can improve firm value significantly. Hall et al. [45] and Cho and Kim [10] also document that exploration can create new market competitiveness for a company and promote its market value. However, over-exploration may also increase the production cost and decrease the product quality of companies [46, 47].

In comparison, extant literature finds the positive effect of equity incentive on R&D investment and patent output, but the effect of vesting periods on corporate innovation is still under debate. As pointed out by Balsmeier et al. [48], innovation strategies are more complex than simple patent. However, the relationship between equity incentive and innovation strategy still remains unclear. Our paper adds to the literature by examining how vesting periods affect innovation strategy. Moreover, although innovation is considered as a long-term project [25], there has been little attention on the impact of equity incentive on the detailed innovation decision-making process, which captures the changes in innovation strategy during the implementation of equity incentive. In this paper, we conduct our research from both cross-sectional and time-series perspectives to perform a comprehensive and overall analysis on the dynamic relationship between vesting period and innovation strategy.

### 2.2. Hypotheses development

#### 2.2.1. Vesting period and exploratory innovation strategy: Cross-sectional analysis

One distinct difference between exploratory and exploitative innovation is the research time, which corresponds to the vesting period of the equity incentive plan. When the vesting period is longer, first, it reduces the short-term performance pressure on executives, shifting their focus from short-term performance to long-term innovation projects. Additionally, it also reduces executive turnover. Innovative projects are designed and led by executives, so their pre-training and familiarity with innovative projects and communication with innovation project teams are invisible human resource costs of innovation projects. When the turnover rate reduces, and the sustainability of innovation investment improves, it is more in line with the characteristics of exploratory innovation. We state our first hypothesis below:

**Hypothesis 1**. From the cross-sectional perspective, the innovation strategy is more exploratory when the vesting period is longer.

#### 2.2.2. Vesting period and exploratory innovation strategy: Time-series analysis

We expect a positive effect of vesting period on exploration in the cross-sectional view, but whether the company chooses to maintain the same innovation strategy as equity incentive plan continues remains to be examined. We expect the innovation strategy may change for at least three reasons. First, Bloom et al. [22] find it usually takes 2–3 years from innovation input to output. Therefore, corporations may shift their innovation strategies from exploratory to exploitative after finishing the major innovation breakthrough because they want to maximize the benefits of the newly completed innovation and enter a “harvest season” [21].

Second, as the internal organizational structure of an enterprise is generally complex, it takes a relatively long time to start or terminate a project for a company [49]. While a reasonable vesting period may provide a natural breakpoint for a failed exploratory innovation and stop loss in time. Third, corporate innovation is highly related to top executives. Aldatmaz et al. [50] show that equity incentive plans can only delay the turnover rate of executives, and the turnover rate increases after three years of the equity incentive plan. Kim et al. [51] also suggest that frequent CEO turnover has a negative impact on firm performance. Overall, during the equity incentive period, the persistency of exploration may not be maintained because of the nature of the innovation project and the company’s personnel or strategy changes. We propose the hypothesis below:

**Hypothesis 2a**. From the time-series perspective, the innovation strategy becomes less explorative and more exploitative after a given time as the equity incentive plan proceeds.
**Hypothesis 2b**. From the time-series perspective, the exploratory innovation strategy intensity maintains stable during the equity incentive plan.

#### 2.2.3. Vesting period and exploratory innovation strategy: Firm size

Firm size may affect the relationship between vesting period and innovation strategy in two ways. First, a large firm usually has an extensive body of complex resources than a small firm, such as human, financial, and social resources [52–54]. Gupta et al. [53] documented that small firms are more restricted in attracting external finance. Therefore, the motivation effect of vesting period might be stronger for large firms due to resource abundance. However, the hierarchies and bureaucratic rules of organizational structure in large firms can also reduce innovation efficiency [55]. Compared to large firms, small firms have greater flexibility and versatility in organizational hierarchical levels, and usually focus on a limited amount of innovation projects, thus facilitate the action from vesting period to innovation strategies. Furthermore, the size advantage and strong market power of large companies may decrease their willingness to engage in exploratory innovation that requires great effort and investment. Thus, we state the following hypothesis:

**Hypothesis 3**. The relation between vesting period and innovation strategy is stronger in small firms.

#### 2.2.4. Vesting period and exploratory innovation strategy: Financial crisis

Besides the firm characteristics, the external environment of firms can also influence the relationship between vesting period and innovation strategy. The global financial crisis that began in 2007 has brought an enormous impact on the global financial market and banking systems [56, 57]. During the financial crisis period, due to the macroeconomic vulnerability, a company’s financial resource, organizational structure, and market environment is unstable and changeable. Therefore, firms may focus more on survival than innovation, weakening the effect of equity incentive on innovation strategy. We state our hypothesis below:

**Hypothesis 4**. The relation between vesting period and innovation strategy is stronger in non-financial crisis period.

## 3. Sample construction and descriptive statistics

### 3.1. Sample selection

Our sample includes all A-shared listed companies in China that have implemented the equity incentive plan from 2006 to 2017, and we obtain 3408 firm-year observations of 1074 companies. Patent and citation information are obtained from the National Bureau of Statistics. Data on equity incentives and other financial information about Chinese listed firms is retrieved from the China Stock Market & Accounting Research (CSMAR) database and Chinese Research Data Services (CNRDS) Platform. We exclude samples with missing values and financial listed companies. All continuous variables are winsorized at the 1st and 99th percentiles.

### 3.2. Measuring exploratory innovation strategy

Following Jia [8], we define exploratory innovation as the degree to which the company’s patent citation use current versus new knowledge, which is measured by citation repetition rate (*Cite*). When the repetition rate is higher, the firm tends to develop and utilize existing patents, and the innovation strategy is less exploratory; when the repetition rate is lower, the enterprise prefers to study new knowledge and technology in unexplored fields and produce more exploratory innovation.

Hall et al. [58] show that the application year can capture the actual time of innovation better than the grating year, so we calculate the citation repetition rate (*Cite*) as the citations in the patent application year divided by the firms existing citation base over the past three years. A firm’s existing citation base includes its previous patent portfolio and the set of patents cited by the firm’s patents filed over the past three years. We also use alternative measures of exploratory innovation for robustness checks.

### 3.3. Measuring vesting duration and control variables

We construct two measures of the vesting period. The first proxy, vesting duration (*Vesting_years*), is defined as the duration of the equity incentive plan. Our second measure of vesting period is vesting phase (*Vesting_phase*), constructed using a set of dummy variables based on each stage of equity incentive.

Following the innovation literature [8, 12], we control for a vector of firm and industry characteristics that may affect a firm’s innovation strategy. Our control variables include firm size (*Size*), profitability (*Roa*), leverage ratio (*Lev*), growth opportunity (*Mb*), equity concentration (*Top1*), the proportion of institutional investor holdings (*Insratio*), the ratio of independent directors (*Inderatio*), board size (*Boardsize*), the nature of property right of actual controller (*Soe*), chairman/CEO duality (*Duality*), firm age (*Age*).

### 3.4. Descriptive statistics

Table 1 reports summary statistics for our sample. The minimum and maximum of the citation repetition rate *Cite* are quite different. The average and standard deviation of citation repetition rate are 3.203% and 6.088%, respectively, indicating that exploratory innovation intensity varies among different firms. On average, a firm implements a 3-year equity incentive plan.

**Table 1:** Summary statistics.

Variables	Mean	S.D.	Min	25th	Median	75th	Max
*Cite*	3.203	6.088	0.000	0.000	0.000	4.386	62.500
*Vesting_years*	3.188	0.540	1.000	3.000	3.000	3.000	5.000
*Vesting_phase*	1.888	0.908	1.000	1.000	2.000	3.000	5.000
*Size*	21.972	1.131	19.699	21.160	21.787	22.592	25.781
*Roa*	5.574	4.878	-23.940	2.910	5.283	8.031	21.322
*Lev*	38.906	18.945	4.777	23.644	38.269	52.302	96.640
*Mb*	2.730	2.080	0.215	1.344	2.171	3.503	12.809
*Top1*	32.469	14.093	8.772	21.609	30.114	41.770	75.005
*Insratio*	35.984	23.496	0.050	15.230	33.953	54.526	86.787
*Inderatio*	37.795	5.682	30.000	33.333	35.714	42.857	57.143
*Boardsize*	2.235	0.176	1.609	2.079	2.303	2.303	2.890
*Age*	6.469	5.252	0.000	3.000	5.000	8.000	27.000
*Soe*	0.110	0.313	0.000	0.000	0.000	0.000	1.000
*Duality*	0.351	0.477	0.000	0.000	0.000	1.000	1.000

This table presents descriptive statistics for innovation and control variables. The table presents mean, median, standard deviation, maximum, and 25^th^ and 75^th^ percentiles for each variable. The sample period spans the period 2006 to 2017. Variable definitions are provided in the S1 File. All continuous variables are winsorized at the 1^st^ and 99^th^ percentiles.

## 4. Main results

### 4.1. Baseline results

We examine the effect of vesting period on a firm’s innovation strategy type using the baseline model as follows:

$$Inno\_type_{i.t}=\beta_{0}+\beta_{1}\times Vesting\;period_{i,t}+\gamma \times Control\;Variables_{i.t-1}+i.Year+i.Industry+\varepsilon_{i.t}$$ (1)

Where *Inno_type*_*i*,*t*_ refers to innovation strategy (*Cite*) of firm *i* in year *t*. The key explanatory variable *Vesting period*_*i*,*t*_ refers to our vesting period measures (*Vesting_years* and *Vesting_phase*) for firm *i* in year *t*. *Control Variables*_*i*,*t-1*_ represents the set of control variables defined in S1 File. As all innovation variables are above zero, we estimate model (1) using the Tobit regression. Ordinary least squares (OLS) regression is also applied in robustness tests. Year and industry fixed effects are included in the model, and we cluster standard errors at firm level.

### 4.2. Vesting period and exploratory innovation strategy: Cross-sectional evidence

Table 2 reports the results of the impact of vesting period on exploratory innovation strategy intensity. It is evident that the coefficients estimate on both *Vesting_years* and *Vesting_phase* are negatively and significantly in all columns. In columns (1), we examine the full sample as specified in Eq. (1) and observe a negative and significant coefficient on *Vesting_years*, suggesting that a one-year increase in the vesting period decreases citation repetition rate by 1.1%, suggesting that exploration innovation intensity rises when the vesting periods are longer. From columns (2) to (5), we partition our sample in each equity incentive phase (the 1st, 2nd, 3rd, 4th, and 5th period of equity incentive plan) and examine the effect of vesting periods on innovation strategy under different phases of equity incentive plans separately. The results in columns (2) to (5) show that the regression coefficients of *Vesting_years* are -0.577, -2.197, -1.847, and -0.400, respectively, indicating that in each equity incentive phase, a one-year increase in the vesting period increases the exploratory innovation intensity. These results complement Liu et al. [12] by taking a further step into the relationship between vesting periods and corporate innovation strategies.

**Table 2:** Vesting periods and exploratory innovation strategy intensity: Cross-sectional results.

	(1)	(2)	(3)	(4)	(5)
	*Cite*	*Cite*	*Cite*	*Cite*	*Cite*
	Vesting duration	Vesting phase			
		1^st^ period	2^nd^ period	3^rd^ period	4^th^ & 5^th^ period
*Vesting_years*	-1.100***				
	(-10.831)				
*Vesting_phase*		-0.577***	-2.197***	-1.847***	-0.400**
		(-4.162)	(-16.486)	(-12.716)	(-2.477)
*Roa*	0.202***	0.079**	0.286***	0.322***	0.171**
	(7.002)	(1.977)	(7.780)	(8.144)	(2.466)
*Lev*	-0.013**	-0.026***	-0.017**	0.026***	-0.315***
	(-2.010)	(-2.991)	(-1.967)	(2.807)	(-24.913)
*Mb*	0.014	0.331***	-0.331***	-0.120	-2.956***
	(0.230)	(4.332)	(-4.002)	(-1.187)	(-15.731)
*Top*1	-0.014*	-0.013	-0.030***	0.012	-0.119***
	(-1.883)	(-1.311)	(-3.032)	(1.137)	(-7.321)
*Insratio*	0.026***	0.034***	0.024***	0.028***	0.013
	(4.638)	(4.608)	(3.495)	(3.740)	(1.164)
*Inderatio*	-0.007	0.002	-0.102***	0.120***	0.261***
	(-0.837)	(0.166)	(-9.219)	(9.886)	(15.096)
*Size*	1.347***	1.736***	0.449***	1.659***	2.316***
	(87.011)	(84.215)	(22.740)	(75.897)	(76.102)
*Soe*	1.536***	1.401***	3.891***	-0.035	-2.472***
	(6.064)	(4.662)	(12.870)	(-0.098)	(-5.535)
*Duality*	-0.923***	-1.597***	-1.069***	0.888***	-4.173***
	(-3.983)	(-4.915)	(-3.612)	(2.672)	(-7.861)
*Age*	-0.099***	-0.147***	-0.018	-0.121***	-0.689***
	(-3.558)	(-4.324)	(-0.527)	(-2.895)	(-9.968)
*Boardsize*	0.642***	2.363***	-1.659***	2.084***	-1.607***
	(4.310)	(11.961)	(-8.721)	(9.832)	(-5.397)
*Year*	Yes	Yes	Yes	Yes	Yes
*Industry*	Yes	Yes	Yes	Yes	Yes
Obs.	1,564	605	515	367	77
Pseudo R^2^	0.015	0.017	0.022	0.038	0.171

This table reports the Tobit regression estimates of vesting period on exploratory innovation intensity using year and industry fixed effects in cross-sectional view. In column 1, all sample firms are included, then samples are partitioned into different equity incentive phase in column 2 to 5. The t-statistics reported in parentheses are based on standard errors clustered by firm. Definitions of variables are provided in the S1 File.

***, **, and * denote significance at the 1%, 5%, and 10% significance levels, respectively.

The coefficients of control variables are generally consistent with prior literature. Enterprises that are older and have simpler organizational structures (CEO duality structure, higher leverage ratio, and higher ownership concentration) are associated with higher exploratory innovation intensity, mainly because they desire radical innovation to change their current performances. While firms that are bigger and state-owned and have a more complicated personnel structure and better operating performance (higher ROA, higher institutional holding ratio, and bigger board) prefer to enjoy their present profit and therefore carry out less exploratory innovation projects. Overall, results in Table 2 support Hypothesis 1 that firms with longer vesting periods are more interested in exploratory innovation.

### 4.3. Vesting period and exploratory innovation strategy: Time-series evidence

We have found the positive relationship between vesting period and exploratory innovation in the above tests, but whether the firm will continue to choose exploratory innovation strategy after breakthroughs during innovation projects still remains to be examined. In this subsection, we examine the time-series effect of vesting periods on exploratory innovation.

Table 3 reports results from regression analyses of the relationship between different vesting phases and exploratory innovation intensity. We define each vesting phase using dummy variables based on the actual vesting phase of equity incentive plan. For example, *1st* equals to 1 if it is in the first period of equity incentive plan and 0 otherwise. In Panel A of Table 3, the coefficients estimate on vesting phase are significantly negative in column (2) and positive in column (4), indicating a turning point in exploratory intensity in the 3rd period. As exploratory innovation takes a relatively long research time, the innovation output begins to produce in the mid-stage of equity inventive period, resulting in a surge in the 3rd period. But in the later stage (after the 4th period), the exploratory innovation intensity falls due to the changes in resource allocation or innovation strategy, which is consistent with González and Xu [21] who find that firms tend to commercialize patents for profit after getting innovation output.

**Table 3:** Vesting periods and exploratory innovation strategy intensity: Time-series results.

Panel A: All sample firms				
	(1)	(2)	(3)	(4)
	*Cite*	*Cite*	*Cite*	*Cite*
2*nd*_*vs*_1*st*	0.133			
	(0.514)			
3*rd*_*vs*_2*nd*		-1.509***		
		(-5.851)		
4*th*_*vs*_3*rd*			-0.040	
			(-0.139)	
5*th*_*vs*_4*th*				2.915***
				(6.855)
*Controls*	Yes	Yes	Yes	Yes
*Year*	Yes	Yes	Yes	Yes
*Industry*	Yes	Yes	Yes	Yes
Obs.	1,120	882	432	76
Pseudo R^2^	0.014	0.019	0.034	0.171
Panel B: Firms with vesting periods bigger than three years				
	(1)	(2)	(3)	(4)
	*Cite*	*Cite*	*Cite*	*Cite*
4*th*&5*th_vs*_2*nd*&3*rd*	1.344***			
	(3.487)			
4*th*&5*th*_*vs*_3*rd*		2.291***		
		(4.914)		
4*th*_*vs*_3*rd*			1.979***	
			(4.241)	
5*th*_*vs*_3*rd*				7.900***
				(15.143)
*Controls*	Yes	Yes	Yes	Yes
*Year*	Yes	Yes	Yes	Yes
*Industry*	Yes	Yes	Yes	Yes
Obs.	217	131	123	76
Pseudo R^2^	0.054	0.085	0.082	0.155

This table reports the Tobit regression estimates of vesting period on exploratory innovation intensity using year and industry fixed effects in time-series view. Panel A reports the difference in the impact of equity incentive on innovation at different stages. Panel B reports the further analyses of motivation effect difference on innovation using firms that has vesting periods longer than three years. The t-statistics reported in parentheses are based on standard errors clustered by firm. Definitions of variables are provided in the S1 File.

***, **, and * denote significance at the 1%, 5%, and 10% significance levels, respectively.

To further examine the changes in exploration intensity around the 3rd period, we use firms with vesting periods longer than three years to examine the relationship between equity incentive and innovation strategy by comparing exploration intensity in different stages of equity incentive. As shown in Panel B of Table 3, we compare the exploratory innovation intensity in different stages of equity incentive, including the comparison between the 4th and 5th periods with the 2nd and 3rd periods, the 4th and 5th periods with the 3rd period, the 4th period with the 3rd period, and the 5th period with the 3rd period. The results show that there is a significant increase in citation repetition rates after the 3rd period. This indicates that although the vesting period motivates corporate to implement more exploratory innovation, the persistency of exploration declines after the 3rd period. Together, the results in Panel A and B of Table 3 support Hypothesis 2a. Therefore, an overlong period may not be able to maintain the exploration intensity in the later stage, only a proper vesting period can play the best role in promoting the exploratory innovation.

### 4.4. Endogeneity issues

We recognize that our baseline results might be biased due to the presence of omitted variables and reversal causality. Although we have controlled for variables that have been identified in the literature as influencing corporate innovation to mitigate this concern, there may still be bias caused by missing variables. The other possible endogenous problem is caused by reversal causality. Equity incentive duration affects exploration intensity; at the same time, the innovation strategy may also influence vesting period. Therefore, we perform two tests to address the endogeneity issues.

Our first approach is the instrumental variable and the results are reported in Table 4. Companies will refer to the previous incentive plans of other companies in the same area when designing equity incentive plans, so the instrument *Years_other* is measured using the average value of vesting period of other companies in the same region (same city) in year *t − 1*.

**Table 4:** Instrumental variable approach.

	1^st^ stage	2^nd^ stage				
	(1)	(2)	(3)	(4)	(5)	(6)
	*Vesting*_*years*	*Cite*	*Cite*	*Cite*	*Cite*	*Cite*
			1^st^ period	2^nd^ period	3^rd^ period	4^th^ & 5^th^ period
*Years_predict*		-6.470***	-9.408***	-5.143***	-7.686***	-5.157***
		(-61.272)	(-64.518)	(-37.984)	(-50.508)	(-25.527)
*Years_other*	0.385***					
	(3.400)					
*Controls*	Yes	Yes	Yes	Yes	Yes	Yes
*Year*	Yes	Yes	Yes	Yes	Yes	Yes
*Industry*	Yes	Yes	Yes	Yes	Yes	Yes
Obs.	2,939	1,493	534	515	367	77
Pseudo R^2^	0.085	0.016	0.020	0.020	0.038	0.173

This table reports the 2SLS regression results using instrumental variable approach. Column 1 reports the estimate of the first-stage regression and columns 2–4 report the estimates of the second-stage regression. The t-statistics reported in parentheses are based on standard errors clustered by firm. Definitions of variables are provided in the S1 File.

***, **, and * denote significance at the 1%, 5%, and 10% significance levels, respectively.

In the first-stage regression, the coefficients on *Years_other* is positive, as expected, and significant at the 1% level, indicating a higher vesting period when other firms have higher vesting durations. In the second stage, the coefficients on the instrumented vesting period are negative, consistent with the baseline results and significant in all columns.

Our second approach is the propensity matching score (PSM) approach. We define firms whose vesting period is higher than three years as long-term incentive groups and short-term incentive groups otherwise. Table 5 reports the results of propensity matching score using eight matching ways. We focus on ATT and find that the cite repetition rate (*Cite*) of short-term incentive groups is about twice as high as that of long-term incentive groups, indicates that long-term equity incentive exceeds short-term equity incentive in promoting exploratory innovation, which is consistent with our baseline analysis.

**Table 5:** Propensity matching score approach.

	Nearest matching (one-to-one)	Nearest matching (one-to-four)	Caliper matching (one-to-one)	Caliper matching (one-to-four)	Kernel matching	LLR (local linear regression) matching	Spline matching	Mahal matching
	m1	m2	m3	m4	m5	m6	m7	m8
	*Cite*							
ATT	1.092***	0.935***	0.898***	0.937***	0.829***	0.995***	0.893**	1.057***
	(2.813)	(2.680)	(2.903)	(2.595)	(2.678)	(2.730)	(2.452)	(3.035)
ATU	0.176	0.924**	0.669**	0.907**	0.665**	0.761**	0.670**	0.638*
	(0.315)	(2.455)	(2.561)	(2.355)	(2.245)	(2.478)	(2.222)	(1.780)
ATE	0.896***	0.933***	0.849***	0.931***	0.794***	0.944***	0.845**	0.974***
	(2.696)	(2.946)	(2.983)	(2.906)	(2.691)	(2.785)	(2.499)	(2.963)
Obs.	1,531	1,531	1,531	1,531	1,531	1,531	1,531	1,564

This table reports the results of propensity matching score. Firms whose vesting periods are lower than three years are categorized as the treatment group, while firms with vesting periods higher than three years are treated as control group. We match firms using eight matching ways on a set of variables that may influence the vesting period, besides, the exploratory innovation intensity, measured by cite repetition rate, in the year before equity incentive is also included in covariates. The t-statistics reported in parentheses are calculated using bootstrap method except the Mahal matching way. Definitions of variables are provided in the S1 File.

***, **, and * denote significance at the 1%, 5%, and 10% significance levels, respectively.

### 4.5. Additional robustness tests

We perform a number of additional tests to ensure that our main results are robust to alternative model specifications and variable definitions. First, define citation repetition rate using the citation base in the previous two years (rather than three years). Second, using as the dependent variable, the citation repetition number instead of citation repetition rate to measure the absolute number of citations repeated. Third, the innovation strategy can be influenced by the company’s previous innovation strategy, so we add the citation repetition rate in year *t − 1* (*L*.*Cite*) to control the impact of past innovation decisions. Fourth, running Ordinary Least Squares (OLS) regression (instead of Tobit regression) to address the robustness of our results. Fifth, using vesting period to classify firms into subsamples that consist of companies with vesting period smaller than, equal to, and bigger than three years, and running partitioning tests using the same model in Table 2. The results of robustness tests are provided in S1 File.

### 4.6. Firm size

We define firm size based on total assets, where firms with total assets higher than the median value are treated as large firms, while firms with total assets below the median value are treated as small firms. In Panel A of Table 6, we find that the impact of vesting period on exploratory innovation is stronger for small firms from the cross-sectional perspective. The evidence is consistent with Bachmann et al. [55] who find that low efficiencies in the decision making process of large companies reduce innovation. Panel B of Table 6 shows the time-series results, the exploration intensity decreases after the 3rd period in small firms and 4th period in large firms. This evidence indicates that from the cross-sectional view, the positive effect of vesting period on exploratory innovation is stronger for small firms. While from the time-series view, the large firms show a more persistent exploratory intensity, which is consistent with the fact that large firms usually have a relatively high level in resource abundance. Together, these results largely support Hypothesis 3.

**Table 6:** Differences in the effects of vesting periods on exploratory innovation intensity: Firm size.

Panel A: Cross-sectional								
	(1)				(2)			
	Small firms				Large firms			
	*Cite*				*Cite*			
*Vesting_years*	-1.932***				-0.266**			
	(-10.853)				(-2.431)			
*Controls*	Yes				Yes			
*Year*	Yes				Yes			
*Industry*	Yes				Yes			
Obs.	762				802			
Pseudo R^2^	0.021				0.019			
p-value	0.000							
Panel B: Time-series								
	(1)	(2)	(3)	(4)	(5)	(6)	(7)	(8)
	Small firms	Large firms	Small firms	Large firms	Small firms	Large firms	Small firms	Large firms
	*Cite*	*Cite*	*Cite*	*Cite*	*Cite*	*Cite*	*Cite*	*Cite*
2*nd*_*vs*_1*st*	0.061	0.205						
	(0.138)	(0.736)						
3*rd*_*vs*_2*nd*			-3.906***	-0.241				
			(-9.056)	(-0.795)				
4*th*_*vs*_3*rd*					0.750	-0.595**		
					(1.476)	(-2.044)		
5*th*_*vs*_4*th*							4.768***	11.664***
							(16.856)	(26.604)
*Controls*	Yes	Yes	Yes	Yes	Yes	Yes	Yes	Yes
*Year*	Yes	Yes	Yes	Yes	Yes	Yes	Yes	Yes
*Industry*	Yes	Yes	Yes	Yes	Yes	Yes	Yes	Yes
Obs.	577	543	410	472	178	254	28	48
Pseudo R^2^	0.022	0.018	0.029	0.025	0.070	0.042	0.609	0.238

This table presents the results of the impact of firm size on the relationship between equity incentive and innovation by partitions firms into subsamples and re-estimates the regression in Tables 2 and 3 for different subsamples. The t-statistics reported in parentheses are based on standard errors clustered by firm. The p-value reported in the last line are based on the seemingly unrelated regressions (SUR) to test the equality of coefficients across subsamples. Definitions of variables are provided in the S1 File.

***, **, and * denote significance at the 1%, 5%, and 10% significance levels, respectively.

### 4.7. Financial crisis

To better capture the influence of the financial crisis in China, we classify the financial crisis period from 2008 to 2011 and split our sample into the crisis period sample and the non-crisis period sample. We present our regression results in Table 7. In Panel A of Table 7, the link between vesting period and exploratory innovation is significant only during the non-financial crisis period from the cross-sectional view. As shown in Panel B of Table 7, in the non-financial crisis period, the exploration intensity declines after the 3rd period during the implementation of equity incentive. However, there is no such trend during the financial crisis period, which indicates that the financial crisis has weakened the motivation effect of vesting period on exploratory innovation. Therefore, Hypothesis 4 holds.

**Table 7:** Differences in the effects of vesting periods on exploratory innovation intensity: Financial crisis.

Panel A: Cross-sectional								
	(1)				(2)			
	Crisis				Non-crisis			
	*Cite*				*Cite*			
*Vesting_years*	-1.028				-1.204***			
	(-0.628)				(-11.534)			
*Controls*	Yes				Yes			
*Year*	Yes				Yes			
*Industry*	Yes				Yes			
Obs.	183				1381			
Pseudo R^2^	0.033				0.015			
Panel B: Time-series								
	(1)	(2)	(3)	(4)	(5)	(6)	(7)	(8)
	Crisis	Non-crisis	Crisis	Non-crisis	Crisis	Non-crisis	Crisis	Non-crisis
	*Cite*	*Cite*	*Cite*	*Cite*	*Cite*	*Cite*	*Cite*	*Cite*
2*nd*_*vs*_1*st*	1.838	-0.239						
	(0.865)	(-0.864)						
3*nd*_*vs*_2*st*			-3.653	-1.315***				
			(-1.190)	(-5.044)				
4*th*_*vs*_3*rd*					-2.083	-0.212		
					(-0.373)	(-0.825)		
5*th*_*vs*_4*th*							\	3.167***
							\	(6.553)
*Controls*	Yes	Yes	Yes	Yes	Yes	Yes	\	Yes
*Year*	Yes	Yes	Yes	Yes	Yes	Yes	\	Yes
*Industry*	Yes	Yes	Yes	Yes	Yes	Yes	\	Yes
Obs.	130	990	89	793	52	380	\	63
Pseudo R^2^	0.042	0.013	0.047	0.020	0.097	0.035	\	0.145

This table presents the results of the impact of financial crisis period on the relationship between equity incentive and innovation by partitions firms into subsamples and re-estimates the regression in Tables 2 and 3 for different subsamples. In column 7, the regression cannot be performed due to insufficient observations. The t-statistics reported in parentheses are based on standard errors clustered by firm. Definitions of variables are provided in the S1 File.

***, **, and * denote significance at the 1%, 5%, and 10% significance levels, respectively.

## 5. Further analysis

We have found that in the cross-sectional perspective, a longer duration is positively related to explorative innovation intensity; while from the time-series view, the explorative innovation intensity shows a downward trend over time, especially after the 3rd period. So how does vesting period affect innovation output? And how do different innovation strategies influence corporate value?

As an important mechanism between equity incentive and innovation projects, innovation strategy will influence innovation output, furtherly reflecting in firm growth [59]. As reported in Table 3, three years is a breakpoint for the consistency of exploratory innovation intensity. In this section, we continue to examine whether innovation output and firm value change when innovation strategy alters.

### 5.1. Equity incentive, innovation strategy, and patent

We start with summary statistics of corporate innovation. Table 8 shows that from the time dimension, there are steady growth trends for innovation output for two-year and three-year equity incentive plans, but a decline in the 3rd period for four-year equity incentive plans.

**Table 8:** Summary statistics of innovation output.

Variables	*Patent*							
Phase	2-year		3-year		4-year		5-year	
1^st^	80.64		77.97		74.99		41.21	
2^nd^	80.45		88.64		88.13		83.11	
3^rd^			95.85		85.65		67.14	
4^th^					32.70		87.33	
5^th^							59.55	
Variables	*Patenti*				*Patentud*			
Phase	2-year	3-year	4-year	5-year	2-year	3-year	4-year	5-year
1^st^	35.66	36.81	30.39	29.95	41.47	38.41	37.15	11.26
2^nd^	42.60	42.67	39.80	44.39	37.45	43.48	42.28	38.72
3^rd^		48.27	31.25	37.50		46.88	44.39	29.64
4^th^			32.70	48.92			41.82	38.42
5^th^				39.00				20.55

This table reports the descriptive statistics of innovation output under different vesting periods and different equity incentive phase. Innovation output is measured using total number of patent applied (*Patent*), total number of invention patent applied (*Patenti*) and total number of utility and design patent (*Patentud*) applied by a firm in a given year. To address the time lag issue in patent output, we use the patent application adjusted by industry and year mean value in year *t* + 1 as the innovation output variable. Detailed definitions of variables are provided in the S1 File.

According to the descriptive statistics in Table 8, different firms under different vesting periods show different innovation growth trends. Therefore, we divided companies into three subsamples, consisting of firms that have vesting periods less than, equal to, and more than three years. Companies with a vesting period equal to three years are used as the benchmark group. The remaining two groups (*Less than 3*, *More than 3*) are used as explanatory variables. The growth trend for patent application is measured using the slope of the line fitted by the corresponding value during equity incentive period. The control variables are the same as defined in Eq. (1).

Following existing literature [3, 60], innovation output is measured using the total number of patent applied (*Patent*), the total number of invention patent applied (*Patenti*) and the total number of utility and design patent (*Patentud*) applied by a firm in a given year. To address the time lag issue in patent output, we use the patent application adjusted by industry and year mean value in year *t+1* as the innovation output variables.

As shown in Table 9, both the growth trends of invention patent application for firms with vesting periods shorter and longer than three years are slower than the three-year equity incentive. Interestingly, the utility and design patent, which represents exploitative innovation, of vesting period over three years increases faster than the three-year equity incentive, indicating that firms prefer to produce exploitation other than exploration after the 3rd period.

**Table 9:** Equity incentive and innovation output: All sample firms.

	(1)	(2)	(3)
	*Patent slope*	*Patenti slope*	*Patentud slope*
*Less than* 3	-0.346	-0.453**	0.194
	(-1.326)	(-2.117)	(1.047)
*More than* 3	-0.192	-0.371*	0.402**
	(-0.715)	(-1.662)	(2.048)
*Controls*	Yes	Yes	Yes
*Year*	Yes	Yes	Yes
*Industry*	Yes	Yes	Yes
Obs.	849	849	849
Adj R^2^	-0.020	0.036	0.028

This table reports the OLS regression estimates of vesting periods on the slopes of innovation output using year and industry fixed effects. The dependent variables are the slopes of innovation output. The t-statistics reported in parentheses are based on standard errors clustered by firm. Definitions of variables are provided in the S1 File.

***, **, and * denote significance at the 1%, 5%, and 10% significance levels, respectively.

To further examine the turning effect around three years, we also report results using samples of companies with vesting periods greater than three years in Table 10. The dependent variable (*Patent growth*, *Patenti growth*, *Patentud growth*) is the growth rate of innovation output in year *t* relative to year *t-1* during equity incentive. The independent variables are whether it is after the 3rd phase *(After the 3rd*) during equity incentive. As shown in the table, patent application falls after the 3rd period, especially exploratory innovation patent (represented by invention patent).

**Table 10:** Equity incentive and innovation output: Firms with vesting periods longer than three years.

	(1)	(2)	(3)	(4)	(5)	(6)	(7)
	*Cite*	*Patent growth*	*Patent growth*	*Patenti growth*	*Patenti growth*	*Patentud growth*	*Patentud growth*
*Cite*			-0.045***		-0.051***		0.013
			(-2.976)		(-3.133)		(0.329)
*After the 3rd*	0.651*	-0.739**	-0.358*	-0.623***	-0.333	-0.608	-0.297
	(1.954)	(-2.549)	(-1.710)	(-2.616)	(-1.511)	(-1.195)	(-0.931)
*Controls*	Yes	Yes	Yes	Yes	Yes	Yes	Yes
*Year*	Yes	Yes	Yes	Yes	Yes	Yes	Yes
*Industry*	Yes	Yes	Yes	Yes	Yes	Yes	Yes
Obs.	271	470	271	432	271	416	237
Adj R^2^	0.047	0.052	0.059	0.015	0.090	0.042	0.091

This table reports the OLS regression estimates of vesting periods on the growth rates of innovation output using year and industry fixed effects. In column 1, the dependent variable is the citation repetition rate, and in column 2–7, the dependent variables are the growth rates of innovation output. The t-statistics reported in parentheses are based on standard errors clustered by firm. Definitions of variables are provided in the S1 File.

***, **, and * denote significance at the 1%, 5%, and 10% significance levels, respectively.

We also use the three-step mediation analysis to examine how the vesting periods affect innovation output through innovation strategy [61]. First, we examine the effect of vesting periods on innovation strategy and results show that exploratory innovation intensity decreases after the 3rd period. Then, we add the mediating variable (exploratory innovation intensity) to the regression of vesting periods to innovation output growth rates. The findings support the mediation effect of innovation strategy in the relationship between vesting periods and innovation output.

### 5.2. Equity incentive, innovation strategy, and firm value

In this section, we focus on the effect of vesting period on firm value. Firm value is measured using the growth rate of Tobin’s Q (*Tobinq growth*) and the slope of Tobin’s Q during the equity incentive plan (*Tobinq slope*).

Table 11 presents the impact of vesting period on firm value. In column (1), the coefficient estimates on *More than 3* is significant and negative, indicating the three-year vesting period is better than other durations in motivating firm value. We then focus on the firms with vesting periods longer than three years. In columns (2), the negative coefficient on *After the 3rd* shows that after the 3rd phase, the growth rate of corporate value reduces. In column (3), the results show that innovation strategy have mediating effects on the relationship between vesting periods and the growth rate of firm value.

**Table 11:** Equity incentive and firm value.

	(1)	(2)	(3)
	All sample firms	Firms with vesting periods longer than three years	
	*Tobinq slope*	*Tobinq growth*	*Tobinq growth*
*Less than* 3	0.380**		
	(2.217)		
*More than* 3	-0.163*		
	(-1.739)		
*After the* 3*rd*		-0.074*	-0.057
		(-1.758)	(-1.579)
*Cite*			-0.010***
			(-4.210)
*Controls*	Yes	Yes	Yes
*Year*	Yes	Yes	Yes
*Industry*	Yes	Yes	Yes
Obs.	1026	641	293
Adj/Pseudo R^2^	0.341	0.471	0.334

This table reports the OLS regression estimates of vesting periods on the slopes and growth rates of firm value using year and industry fixed effects. In column 1, the dependent variable is the slope of Tobin’s Q, and in column 2–3, the dependent variables are the growth rates of Tobin’s Q. The t-statistics reported in parentheses are based on standard errors clustered by firm. Definitions of variables are provided in the S1 File.

***, **, and * denote significance at the 1%, 5%, and 10% significance levels, respectively.

## 6. Summary and conclusion

In this paper, we examine the impact of the vesting period of equity incentive plan on corporate innovation strategy. Using a sample of Chinese listed firms from 2006 to 2017, we find that firms with longer vesting periods are associated with more exploratory innovation strategies in both cross-sectional and time-series positions. However, exploratory innovation intensity decreases as the equity incentive plans implements. These results are robust to a variety of tests on model specification, variable definitions, and endogeneity issues. Moreover, we find that the positive effect of vesting period on exploratory innovation is more pronounced in small firms and during the non-financial crisis period. Further analyses suggest that the growth rates of innovation output and firm value decrease, especially after the 3rd period of equity incentive plan. This means that an excessively long duration can lead to a lack of innovation motivation in the later stage and impede firm development.

Our paper shed new light on the importance of vesting period, a key factor in equity incentive plan, and its effect on innovation strategy. Despite abundant studies on the relationship between equity incentive and innovation, few literature examines the role of vesting period in the innovation decision-making process. In particular, our findings enhance our understanding of companies’ preference for exploratory innovation under different vesting periods and in different phases, thus enriching the literature on corporate innovation by providing evidence from the dynamic innovation decision-making process. Our findings also discover that an over-long vesting period can hinder firm performance. Therefore, it is critical for firms to choose the appropriate vesting period that maximizes the motivation effect of equity incentive on exploration and firm value. A useful direction for future research would be to study the impact of other factors in equity incentive on innovation strategy. And another fruitful direction for future research is to find the proper incentive scheme that balances exploration and exploitation, thus to maximize the motivation effect of equity incentive and achieve the profit maximization goal for companies.

## Supporting information

S1 File Variable definition and robustness checks. (DOCX)
