# Supplementary material for: Exploration or exploitation? A study on equity incentive design, dynamic decision making, and economic consequences
Source: PLoS One. 2023 Jan 23;18(1):e0277965. doi: 10.1371/journal.pone.0277965 (PMC9870139; doi:10.1371/journal.pone.0277965)
Supplement: S1 File — (DOCX) [file pone.0277965.s001.docx]

**Appendix A. Variable definition**

| Variable | Definition |
| --- | --- |
| $Cite$ | The citations in the patent application year divided by the firms existing citation base over the past three years |
| $Patent$ | the total number of patent applications by a firm in a year t + 1 |
| $Patenti$ | the total number of invention patent applications by a firm in a year t + 1 |
| $Patentud$ | the total number of utility model and design patent applications by a firm in a year t + 1 |
| $Vesting\_years$ | the duration of the equity incentive plan |
| $Vesting\_phase$ | A set of dummy variables based on different phase of the equity incentive plan |
| $Size$ | The natural logarithm of the book value of total assets in year t |
| $Roa$ | Return on total assets |
| $Lev$ | Leverage ratio, defined as total liabilities divided by total assets |
| $Mb$ | Mark-to-book ratio, equaling market value of equity divided by the book value of total assets |
| $Top1$ | Ownership concentration, calculated as the biggest shareholder’s stock holdings divided by the number of A shares issued |
| $Insratio$ | Institutional investors holding shares divided by the number of outstanding shares |
| $Inderatio$ | Number of independent directors divided by the total board members |
| $Boardsize$ | The natural logarithm of total board members |
| $Soe$ | An indicator variable that equals to 1 for stated-owned enterprises and 0 otherwise |
| $Duality$ | An indicator variable that equals to 1 if the CEO and chairman are the same person and 0 otherwise |
| $Age$ | Number of years since the firm’s IPO |

**Appendix B. Additional robustness tests**

Additional robustness test 1: alternative variable definition

|  | | (1) | (2) | (3) | (4) | (5) |
| --- | --- | --- | --- | --- | --- | --- |
|  | |  | Phase 1^st^ | Phase 2^nd^ | Phase 3^rd^ | Phase 4^th^ &5^th^ |
| Panel A | $Cite\_2$ | | | | | |
| $Vesting\_years$ | | -0.658*** | -0.265** | -1.589*** | -1.611*** | 1.143*** |
|  | | (-6.793) | (-2.008) | (-12.055) | (-12.345) | (7.048) |
| $Controls$ | | Yes | Yes | Yes | Yes | Yes |
| $Year$ | | Yes | Yes | Yes | Yes | Yes |
| $Industry$ | | Yes | Yes | Yes | Yes | Yes |
| Obs. | | 1,584 | 607 | 526 | 367 | 77 |
| Pseudo R^2^ | | 0.015 | 0.019 | 0.021 | 0.037 | 0.018 |
| Panel B | $Cite\_absolute$ | | | | | |
| $Vesting\_years$ | | -11.306*** | -1.501 | -13.166*** | -18.061*** | 8.666*** |
|  | | (-5.378) | (-0.659) | (-6.846) | (-5.216) | (20.704) |
| $Controls$ | | Yes | Yes | Yes | Yes | Yes |
| $Year$ | | Yes | Yes | Yes | Yes | Yes |
| $Industry$ | | Yes | Yes | Yes | Yes | Yes |
| Obs. | | 1,685 | 647 | 568 | 391 | 79 |
| Pseudo R^2^ | | 0.024 | 0.038 | 0.031 | 0.040 | 0.165 |

This table reports the robustness check results using regression in Table 2 after changes of variable definition. The dependent variable in Panel A is the citation repetition rate using the citation base in the previous two years (rather than three years). The dependent variable in Panel B is the citation repetition number (rather than citation repetition rate). The t-statistics reported in parentheses are based on standard errors clustered by firm. Definitions of variables are provided in the Appendix A. ***, **, and * denote significance at the 1%, 5%, and 10% significance levels, respectively.

Additional robustness test 2: adding a one-period lagged variable

|  | (1) | (2) | (3) | (4) | (5) |
| --- | --- | --- | --- | --- | --- |
|  | $Cite$ | $Cite$ | $Cite$ | $Cite$ | $Cite$ |
|  |  | Phase 1^st^ | Phase 2^nd^ | Phase 3^rd^ | Phase 4^th^ &5^th^ |
| $Vesting\_years$ | -0.517*** | -0.775*** | -1.959*** | -0.808 | -1.863* |
|  | (-7.052) | (-6.835) | (-15.991) | (-0.661) | (-1.701) |
| $L.Cite$ | 0.595*** | 0.686*** | 0.569*** | 0.528*** | 0.945*** |
|  | (50.194) | (36.990) | (33.938) | (4.195) | (4.372) |
| $Controls$ | Yes | Yes | Yes | Yes | Yes |
| $Year$ | Yes | Yes | Yes | Yes | Yes |
| $Industry$ | Yes | Yes | Yes | Yes | Yes |
| Obs. | 1,324 | 521 | 425 | 316 | 62 |
| Pseudo R^2^ | 0.037 | 0.047 | 0.045 | 0.046 | 0.243 |

This table reports robustness check results using regression in Table 2 after adding the one-period lagged citation repetition rate ($L.Cite$) to control variables. The t-statistics reported in parentheses are based on standard errors clustered by firm. Definitions of variables are provided in the Appendix A. ***, **, and * denote significance at the 1%, 5%, and 10% significance levels, respectively.

Additional robustness test 3: OLS regression

|  | (1) | (2) | (3) | (4) | (5) |
| --- | --- | --- | --- | --- | --- |
|  | $Cite$ | $Cite$ | $Cite$ | $Cite$ | $Cite$ |
|  |  | Phase 1^st^ | Phase 2^nd^ | Phase 3^rd^ | Phase 4^th^ &5^th^ |
| $Vesting\_years$ | -0.539* | -0.296 | -0.989** | -0.773 | -0.977 |
|  | (-1.661) | (-0.728) | (-2.015) | (-1.430) | (-0.693) |
| $Controls$ | Yes | Yes | Yes | Yes | Yes |
| $Year$ | Yes | Yes | Yes | Yes | Yes |
| $Industry$ | Yes | Yes | Yes | Yes | Yes |
| Obs. | 1,564 | 605 | 515 | 367 | 77 |
| Adj R^2^ | 0.020 | -0.007 | 0.002 | 0.016 | 0.109 |

This table reports robustness check results using OLS regression in Table 2. The t-statistics reported in parentheses are based on standard errors clustered by firm. Definitions of variables are provided in the Appendix A. ***, **, and * denote significance at the 1%, 5%, and 10% significance levels, respectively.

Additional robustness test 4: partitioning tests

|  | (1) | (2) | (3) |
| --- | --- | --- | --- |
|  | $Cite$ | $Cite$ | $Cite$ |
|  | Vesting periods  less than 3 years | Vesting periods  equal to 3 years | Vesting periods  more than 3 years |
| $Vesting\_phase$ | 0.606** | -0.614*** | -0.203 |
|  | (2.400) | (-3.812) | (-1.303) |
| $Controls$ | Yes | Yes | Yes |
| $Year$ | Yes | Yes | Yes |
| $Industry$ | Yes | Yes | Yes |
| Obs. | 60 | 1,193 | 311 |
| Pseudo R^2^ | 0.131 | 0.018 | 0.035 |

This table reports robustness check results using regression in Table 2 after split the whole sample into three groups based on vesting periods. The t-statistics reported in parentheses are based on standard errors clustered by firm. Definitions of variables are provided in the Appendix A. ***, **, and * denote significance at the 1%, 5%, and 10% significance levels, respectively.
